# Seasonal methane emission from municipal solid waste disposal sites in Lagos, Nigeria

**DOI:** 10.1038/s41598-022-22923-5

**Published:** 2022-10-31

**Authors:** H. S. Riman, G. U. Adie, W. U. Anake, G. R. E. E. Ana

**Affiliations:** 1 Department of Chemistry, Faculty of Science, University of Ibadan, Ibadan, Oyo State Nigeria; 2 Department of Chemistry. College of Science and Technology, Covenant University, Ota, Ogun State Nigeria; 3 Department of Environmental Health Sciences, Faculty of Public Health, College of Medicine, University of Ibadan, Ibadan, Oyo,State Nigeria

**Keywords:** Climate sciences, Chemistry

## Abstract

The Municipal Solid Waste (MSW) Sector is a major source of Methane (CH_4_) emission, a Greenhouse Gas (GHG) that contributes to Climate Change. However, governments of developing countries have not been able to address the challenges posed by this sector due to inadequate funding and technical requirement. The objective of this study was to determine how seasonal variation influences the CH_4_ gas emission. The First Order Decay (FOD) Tier 1 Model was used to estimate CH_4_ emission from four Solid Waste Disposal Sites (SWDS) in Lagos namely: Ewu-Elepe (Ewu), Abule-Egba (A/E), Soluos (Sol), and Olushosun (Olu) covering the dry and wet seasons, respectively for the inventory year 2020. A known weight of the wet waste deposited was characterized. The study revealed that the Degradable Organic Carbon (DOC) for the dry season was 12.897 GgC/kgWaste while that of the wet season was 12.547 GgC/kgWaste. But, the methane gas generated during the wet season was 0.331 Gg higher than that of the dry season which was 0.134 Gg for the study period. This is an appreciable quantity of methane that can contribute to the global Climate Change impact if not addressed. Therefore, these waste types should be segregated from other recyclables and processed into compost or energy resource.

## Introduction

The Municipal Solid Waste (MSW) Sector has been identified by the United States Environmental Protection Agency (US EPA) and Intergovernmental Panel on Climate Change (IPCC) as a major source of methane (CH_4_) gas emission. CH_4_ is a greenhouse gas that has a global warming potential of 21 times more than carbon dioxide (CO_2_), a leading cause of Climate Change^1–3^. MSW is specified as waste generated from households, markets, restaurants, public institutions, industrial installations, sewage facilities, agricultural activities, construction, and demolition sites which most often are mixed in a waste stream^4,5^.

Lagos is a commercial hub and one of Africa’s most populous cities. Rapid population growth has led to a rise in the volume of MSW being generated within the city metropolis. The megacity has an estimated population of over 15 million people located in southwest Nigeria^6^**.** In the past few decades, Lagos State has witnessed an increase in urban and semi-urban population surges due to migration and industrialization. The management and disposal of MSW in Lagos State is mostly been carried out by the Lagos State Waste Management Authority (LAWMA) as the regulatory agency and Registered Private Sector Participation (PSP). The State's waste management practices comprises waste generation, collection, and transportation to Solid Waste Disposal Sites (SWDS) located within the city metropolis^7,8^. However, MSW composition is the main factor influencing emissions from solid waste disposal sites. Other factors include cultural norms, living standards, economic growth, climate, and energy consumption^9–11^.

According to United Nations Environment Program (UNEP) classification, the municipal solid waste stream comprises organic waste types such as food waste, garden/yard waste, paper, and wood products, while the inorganic waste type includes; plastic, metal, glass, and electronic waste. These waste types constitute the bulk of the solid waste and differ in size and texture^12–14^. However, organic waste undergoes degradation depending on the waste type. Some degrade rapidly, others moderately, whereas some waste degrades slowly. Hence, the Degradable Organic Carbon (DOC) is one of the main factors affecting the amount of CH_4_ emission from SWDS and is estimated based on the waste composition and varies for different waste fractions^15,16^. Although, the climatic conditions of the SWDS also influence the anaerobic decomposition of organic waste. However, MSW degradation varies for different waste types: For example, Food waste, Grasses/Garden waste degrades rapidly, whereas Paper/Textile/Nappies waste degrades moderately, the Wood and Wood products degrade slowly, while Metals, Nylons, Leather, and Plastic are non-degradable waste^17^. Also, the choice of degradation time usually depends on the physical and chemical conditions in the SWDS such as the waste's temperature, humidity, and chemical composition of the waste^18,19^. Furthermore, the temperature depends on the season, during the dry season, the temperature is > 20 °C, and at this temperature, CH_4_ is generated but is subsequently converted to heat energy resulting in unintentional/uncontrolled fire outbreaks in the SWDS^2,3^.

Some researchers who carried out similar studies in Lagos SWDS are: Aboyade^20^ used a system dynamic model in his studies at Olusosun dumpsite to determine the interaction between MSW composition, energy, and climate change. Oyelola and Babatunde^21^ determined the MSW composition of households and markets in Lagos, while Balogun-Adeleye et al.^7^ used US-EPA Landfill Gas Emission Model (LANDGEM) to determine methane generation potential in Olushosun landfill, Lagos. Despite these studies being conducted, there is still no data on the Degradable Organic Carbon (DOC) and the quantity of CH_4_ emitted based on seasonal variation. Therefore, this study aims to estimate the amount of DOC in the MSW and determine the CH_4_ emission based on seasonal variations using the First Order Decay (FOD) Tier 1 model for the inventory year 2020.

## Materials and methods

### Site description and survey period

**Figure 1:**
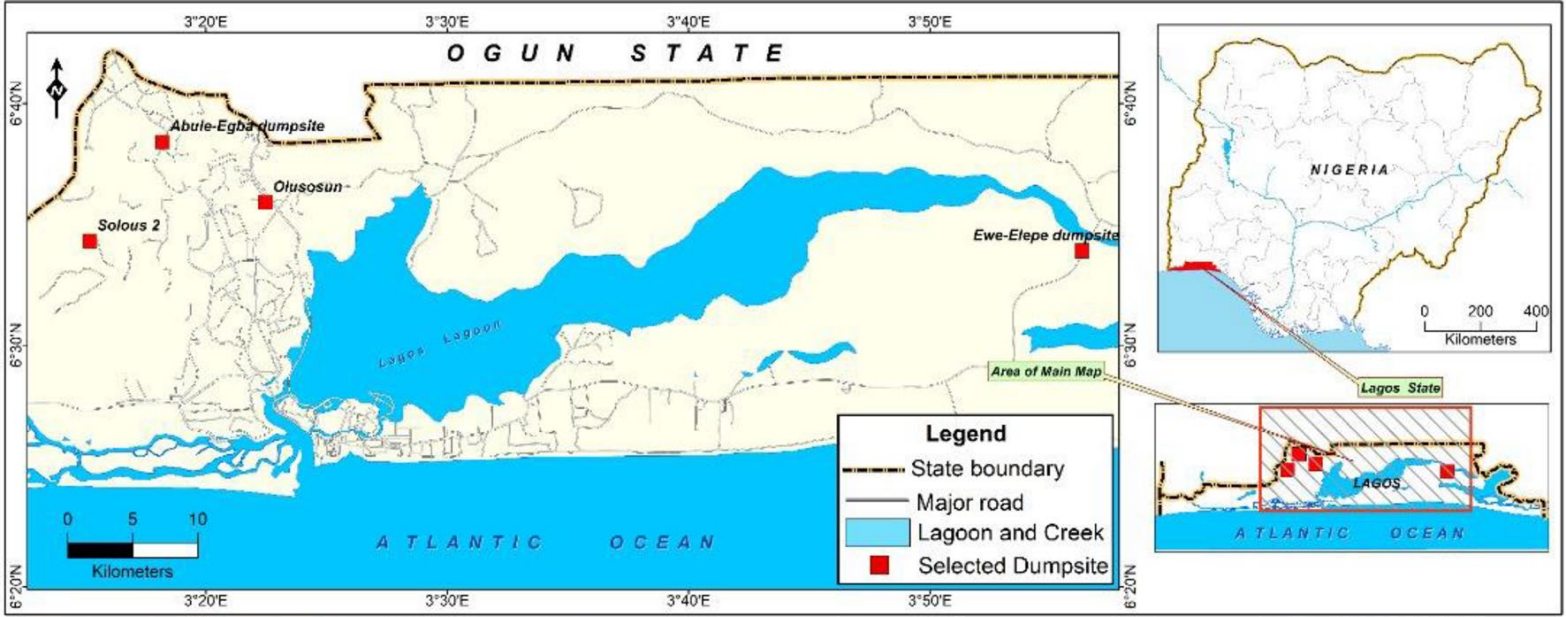
Map of Lagos showing the selected Solid Waste Disposal Sites (Dumpsites). (Source: ESRI 2020. ArcGIS Desktop: Release 10.8 Redlands, CA: Environmental Systems Research Institute).

Lagos State sprawls inland from the Gulf of Guinea situated on Latitude 6° 27′55.5192N and Longitude 3° 24′23.2128E. The city of Lagos is divided into the mainland and the Island areas, comprising residential, institutional, industrial, and commercial settlements. The SWDS that are located within the mainland are Abule-Egba, Solous 2 and Olushosun dumpsites while that of the Island area is the Ewe-Elepe SWDS along Lekki-Epe Road as shown in (Fig. 1). The survey and sampling were done between January to March 2020 for the dry season and May to July 2020 for the wet season.

### Waste characterization

The solid waste characterization was done by random sampling within the active site of each SWDS. At each dumpsite, 5 locations within the active site where chosen and 5 kg of the solid waste was collected and mixed to obtain 25 kg of the wet waste using a weighing balance and then sorted to determine the different waste types and their quantities in the bulk waste. The physical conditions at the SWDS such as shallow depths of < 5 m and limited soil cover (capping) were observed. Also, fire incidences were a regular occurrence during the dry season along with active scavenging activities. Other significant features of the SWDS are shown in (Table 1).

**Table 1:** Features of SWDS in Lagos.

Features	Solid waste disposal site			
	Ewu-Elepe (EWU)	Abule-Egba (A/E)	Solous (SOL)	Olushosun (OLU)
Location	Lekki/Epe Rd	Lagos/Abeokuta Rd	Ishire, Lasu Rd. Igundo	Ojota-Ikeja
Size (hectares)	20	10.2	12.1	42.7
Capacity for use (%)	30	35	30	40
Life span (year)	10	15	10	20
Average collection per day (T)	1864	2250	1000	10,000
Dumping quality	Open	Open	Open	Open
Waste management. facility	A	NA	A	NA
LFG collection system	NA	NA	NA	NA

*NA* not available, *A* available (can recycling facility), *LFG* landfill gas.

Expected waste management facilities are: weighing balances, incinerating and recycling facilities.

Source: LAWMA, 2019.

### Determination of total quantities of MSW brought into SWDS

MSW is disposed of daily as collected from different locations using waste collection trucks. All the SWDS are located within the city metropolis in residential areas except the Ewu-Elepe which is on the outskirt of city. Of these sites, Ewu-Elepe SWDS had a weighing balance for daily measurement of solid waste brought into the site but at the time of this field investigation, the weighing balance was no longer in use. Hence, the amount of waste brought to the facilities was determined by the size of the truck in tons and the number of trucks per day. The trucks were usually 9, 11, and 14 tons of 15 to 20 trips per day. Each location received 15–20 trucks of 11 tons weighted average waste per day. A field investigation was carried out within four consecutive days in a month for 3 months per season. Each SWDS was visited six times within the sampling period. A total visit of 24 times was carried out to four SWDS for both seasons. At each SWDS, a known weight of solid waste was sorted to determine the waste composition.

### Estimation of methane emission from MSW

Methane emission generation potential is usually calculated from the MSW disposed of at SWDS based on the waste type and the waste management practice at the disposal sites^22^. Several methods have been used for the estimation of CH_4_ emission from SWDS. Some popular ones include the IPCC default method used by Singh et al^3^, FOD Model Tier 1 and Tier 2 used by Jeong^23^. The Land GEM Model was used by Balogun-Adeleye et al^7^ other methods are: Modified Triangular Method (MTM), Close Flux, and In-Situ Measurement Method. The FOD Tier 1 model to estimate methane emission was adopted for this study because it accommodates a seasonal variation of the degradation process for estimating annual emission. Furthermore, it determines the amount of the gas emission as the fugitive emission from the landfill surface to the air and requires information on the waste composition and physical conditions at the SWDS^24^.

### Methane emission equations

1 $$DOC = 0.4\left( A \right) + 0.17\left( B \right) + 0.15\left( C \right) + 0.30\left( D \right)$$

DOC is the fraction of degradable organic content in waste type obtained from the waste characterization.

where: A = Paper waste; B = Grasses/Garden Waste; C = Food Waste; D = Wood waste

2 $$DDOC_{mdt} = W_{i} *DOC*DOC_{f} *MCF$$

DDOC_m_ = the mass of decomposable degradable organic carbon in the waste, DDOC_mdt_ = the mass of decomposable degradable organic waste deposited for the wet and the dry season.

where: W_i_ = Weight of wet waste for dry and wet seasons (Table 2), DOC = Degradable Organic Carbon.

DOC_f_ = Fraction of DOC that can decompose and be converted to methane, MCF = Methane correction factor for aerobic decomposition in the year of deposition

3 $$DDOC_{mat} = DDOC_{mdt} + \left( {DDOC_{maT - 1} *e^{ - k} } \right)$$

DDOC_mat_ = The mass of decomposable degradable organic carbon that accumulated in the SWDS at the end of a Season. DDOC_mdt_ = The mass of decomposable degradable organic waste deposited for the wet and the dry season.

Where: DDOC_maT-1_ = Total mass of accumulated decomposable organic waste for both seasons added together.

K = The decay constant of the decay process.

4 $$DDOC_{mdecompT} = DDOC_{maT - 1} *\left( {1 - \exp^{ - k} } \right)$$

DDOC_mdecompt_ = the Decomposable Degradable waste that has decomposed at the SWDS

5 $$CH_{4} \; Generated = DDOC_{mdecompT} *F*16/12$$

CH_4_ Generated = The methane generated from the fraction of the decomposed organic carbon based on the methane to carbon ratio in the SWDS.

Where: F = Percent of organic waste being converted to methane, 16/12 = Methane to Carbon ratio

6 $$CH_{4} \; Emission = \sum {\left[ {CH_{4} \; Generated - R_{x} } \right]} *\left( {1 - OX_{T} } \right)$$

CH_4_ Emission is the methane generated from the SWDS and oxidized into the atmosphere when no methane was recovered.

Where: R_X_ = Recovered methane; OX_T_ = Oxidation factor in year T.

The following parameters and the default values used are:

K = 0.065 (Dry season), 0.17(Wet season); F = 0.5; R_x_ = 0; OX = 0; MCF = 0.4; DOC_f_ = 0.5 and T = 2020, inventory year^2^.

## Results

### Waste characterization in each SWDS per month

Waste characterization is a major waste management strategy. It is one of the ways to achieve sustainable development while reducing emissions. The amount of waste deposited is used to estimate landfill gas emissions; based on field measurements^25^. The monthly and seasonal scale variability in weather conditions leads to wet and dry seasons of various environmental extreme events like drought, heat waves, floods, snowmelt, and global warming with a severe negative impact on humans and the ecosystem according to Sreeparvathy and Srinivas^26^. Table 2 shows the organic waste types identified in SWDS during sampling period such as; Food waste, Paper/Textile, Grasses/Garden waste, and Wood waste.

### Seasonal variation of waste deposited at SWDS

The waste characterization as shown in (Table 2**)** above revealed that the same type of organic waste was being disposed of across the four SWDS in different quantities. It was observed that A/E SWDS had the highest value of food waste when compared to other SWDS and also, food waste has the highest values among other SWDS when compared to other waste types. Food waste and grasses/garden waste have shown an increase in value for the dry season while paper/textile and wood wastes had an increase in value for the wet season. The grasses/garden waste has a significant increase of 29.6% during the dry season above the wet seasons. The percentage average of organic waste disposed of for the dry season was 54. 6% while 50.6% was disposed of during the wet season.

**Table 2:** Wet weight of organic waste deposited for both dry and wet seasons in kilogram (kg).

	Dry season												
	Jan 2020				Feb 2020				March 2020				Wi
Location:	EWE	A/E	SOL	OLO	EWE	A/E	SOL	OLO	EWE	A/E	SOL	OLO	
**Waste types**													
Food waste	30.1	30.0	25.4	25.1	25.1	30.6	21.7	25.9	20.4	27.5	24.1	21.7	307.6
Paper/textile	15.3	20.2	20.7	22.3	14.3	12.0	16.5	15.7	13.1	12.7	14.7	13.3	190.8
Grasse/garden	7.8	5.9	8.3	7.5	10.3	11.2	9.9	7.3	12.1	7.3	15.6	10.2	103.2
Wood	2.0	2.1	4.5	4.5	1.8	5.8	6.5	6.1	2.3	2.6	2.5	3.2	43.9
Total													645.5

	Wet season												
	May 2020				June 2020				July 2020				
**Waste types**													
Food waste	25.4	23.6	26.9	22.7	23.9	25.9	22.7	23.0	22.5	30.4	25.1	20.5	292.6
Paper/textile	13.1	17.1	12.7	15.1	15.2	20.7	22.9	17.5	14.5	20.3	17.6	15.2	201.9
Grass/garden	6.0	3.8	4.2	4.9	4.7	3.2	3.8	4.2	6.3	3.7	9.2	7.3	61.3
Wood	4.3	3.6	5.7	5.3	5.6	5.0	3.1	4.0	2.1	3.1	5.0	4.9	51.7
Total													607.5
Overall Total													1253

(Table 3**)** shows the stepwise degradation process of organic waste that leads to the generation of CH_4_ gas. The DOC values were obtained from Eq. (1). After the percentage average of each waste type was estimated. The DOC value for the dry season was seen to be higher than that obtained during the wet season.

**Table 3:** Results for DOC, DDOC_mdt_, DDOC_maT_, DDOC_mdecompT_ and CH_4_ generated.

Result parameters	Seasons	
	Dry	Wet
DOC (GgCarbon/kgWaste)	12.897	12.547
DDOC_mdt_ (GgC/kgW)	1.665	1.524
DDOC_maT_ (GgC/kgW)	4.653	4.216
DDOC_mdecompT_ (GgC/kgW)	0.201	0.497
CH_4_ Generated (Gg)	0.134	0.331

The DDOC_m_ was calculated based on (Eq. 2). It is the mass of organic carbon that will degrade under the anaerobic conditions in the SWDS. The DDOC_mdt_ is used to indicate the period under which the degradation took place, which reflects the dry and wet seasons as used in this study. The value is obtained based on factors such as the weight of the wet waste (Wi), the waste type, the physical conditions of the SWDS, and the weather conditions as shown in Table 3. However, the dry season showed a value of 1.665 GgC/KgW which is higher when compared with the wet season value of 1.524 GgC/KgW.

The DDOC_maT_ was applied based on (Eq. 3). It was used to determine the accumulated degradable waste over a period which has shown a 4.93% value higher for the dry season over the wet season.

The DDOC_mdecompT_ was applied on (Eq. 4). It is the decomposable degradable organic carbon material that has undergone anaerobic processes by micro-organisms under particular weather conditions of temperature. The wet season value was 0.497 GgC/KgW which is higher than 0.201 GgC/KgW decomposed during the dry season. Hence, the CH_4_ generated for the wet season was 0.331 Gg while the dry season value was 0.134 Gg. This shows a 42.2% increase in methane generation in the wet season above the dry season with consideration of the conversion factors and the methane to carbon ratio as applied in (Eq. 5).

From (Eq. 6) the CH_4_ generated is equal to the CH_4_ being emitted because there was no methane gas recovery for energy purpose and the oxidation value for open dumpsites is zero by default^2^.

The statistical tool used to compare the relationship between MSW composition across SWDS with seasonal variation was IBM SPSS Statistics 16.0. Correlation coefficient was carried out for each waste type and the data for dry season (X) and wet season (Y) were compared. The result showed that Food waste (r)value was 0.5 which is positively correlated. Grasses/Garden (r) value was 0.67 which is positively correlated and significant at 0.05 levels, while Wood waste (r) value was 0.056 showing that it is weakly correlated. However, Paper/Textile (r) value was − 0.31 which is negative, showing little or no correlation of waste composition across SWDS with seasonal variation,

## Discussion

### Municipal solid waste composition

MSW has become a global environmental concern. The contribution of MSW sector to the emission of GHG into the atmosphere has caused negative impacts. The impact of these gases especially methane has led to Climate Change which has further affected other aspects of the ecosystem and public health. For example, coral reefs in the marine habitat have been observed to be undergoing stress and subsequent reduction in population as reported by Chimienti et al.^27^. Also, Parihar et al.^28^ has revealed the effect of Climate Change on malaria transmission due to the potential increase in mosquito density as a result of increase in extreme weather condition of temperature and rainfall as one of the health effects of GHGs. The composition of MSW in countries like India showed a 40–60% organic waste content as reported by Mathur et al^4^. While other findings by Srinivasan et al.^16^ in Chennai City, India the values is 54% of degradable organic waste. Also, 75% of organic waste was reported by Hosseini et al.^29^ in Iran. While the value of degradable waste for this study is 52.6%. But in Poland, which is a developed country, the value of 41.1% biodegradable waste was reported by Ciula et al.^24^. The reason why the value of organic waste from MSW in developing countries is higher than in developed countries could be because, in developing countries, there is no waste segregation to various recycling facilities at the point of waste generation, collection, and transportation as is the case with developed countries. Also, in developed countries, some waste such as cans, plastics, nylons, and cartons are directly transported to recycling facilities. Hence, the waste Collection Coverage (CC) for developed countries is 100%, whereas; the waste CC for developing countries is 40–50% as stated by Froiland-Jesen and Pipatti^30^. Although, the waste CC for Lagos is quite effective because the waste generated is usually being collected and transported to SWDS by the relevant government agency and PSP. Unfortunately, not all the waste generated is being collected and disposed of due to bad roads and breakdown of the waste trucks. However, some are been dumped in drainage systems or on unauthorized locations.

### The waste types deposited in SWDS

The MSW generated and subsequently deposited in Lagos SWDS within the city metropolis comprises rapidly degradable, moderately degradable, slowly degradable, and non-degradable waste**.** Hence, the difference in the quantity and type of waste deposited in each SWDS is thought to be influenced by the population and economic activities within the location. The reason for the low value of the degradable waste disposed of during the wet season as shown in (Table 2) could be as a result of the degradation process which begins to take place in the waste bins before the arrival of the waste truck since the waste is sometimes not being collected on daily basis. As shown in (Table 4), The IPCC values for West Africa and Nigeria are quite different from the values obtained from this study even though, some waste types are within a close range of values. In addition, the results reported by^7,21^ have shown that previous Lagos values are also different from the results obtained from this study. This discrepancy could be due to the fact that: during this study sampling and field investigations across the major SWDS was conducted within the last week of the months. This period is fixed by the State LAWMA to compulsorily carryout environmental sanitation when general environmental clean-up exercises are enforced by the State Government.

**Table 4:** Percentage weight of MSW for previous studies and this study.

	IPCC 2019 default values		Lagos values		
	West Africa	Nigeria	Oyelola and Babatunde, 2008	Balogun-Adeleye, 2018	This study
Food waste	53.9	63.6	68.16	50.8	25.0
Grasses/garden	NA	NA	4.20	NA	7.2
Paper	9.4	11.3	12.46	32.4	16.4
Plastic	6.4	8.7	3.64	6.7	9.3
Leather	NA	NA	NA	NA	8.0
Metals	2.7	3.2	2.08	4.2	3.6
Glass	1.3	2.6	1.78	1.78	NA
Others	26.5	10.6	7.68	2.8	30.0

NA not available.

Previous research results and this study have shown that food waste has the highest value when compared to other waste types. Also, this study has revealed an appreciable value of 7.2% for Grasses/Garden waste while other studies reported zero value, although Oyelola and Babatunde^21^] indicated the waste category as 4.2%. This difference could be as a result of the sampling period which resulted in an increased volume of grasses, and garden/yard waste being disposed of in SWDS after general environmental clean-up exercise which usually involves cutting of yard grasses and trimming of flowers. The percentage of degradable waste in the total waste stream was 52.6% in this study but Balogun-Adeleye et al^7^ indicated 83.2%, and Oyelola and Babatunde^21^ reported 90.78%. However, Ciula et al^24^ in their findings conducted in Poland kept the value of vegetables, paper, and cardboard waste at 14.5% . This value is small confirming what Mathur et al^4^ stated that the composition of degradable Municipal Solid Waste in developed countries is low while that of developing countries is about 40–60%.

### The degradable organic carbon (DOC)

**Figure 2:**
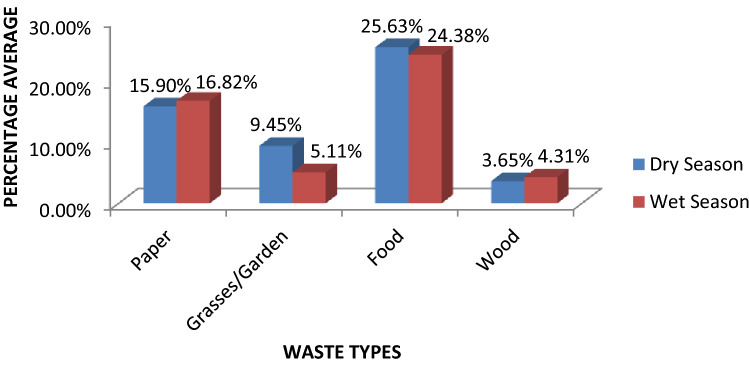
Percentage average of each organic waste type disposed of per season.

One factor that determines methane generation in SWDS is the amount of Degradable Organic Carbon (DOC) in the waste stream, which depends on the waste composition as shown in (Figure 2). DOC is one of the main parameters affecting the CH_4_ emission from SWDS. It is observed to correlate with the quantity of degradable waste deposited, and the physical conditions at the SWDS which leads to the formation of Decomposable Degradable Organic Carbon (DDOC). Meanwhile, the Mass of Decomposable Degradable Organic Carbon (DDOCm) from the waste deposited and accumulated was higher during the dry season than during the wet season because, waste Collection Coverage (CC) was more effective during the period due to the trucks ability to access various waste collection routes. In Poland, Ciula et al.^24^ used the First Order Decay Model and the DOC value obtained was 0.061 GgC/Ggwaste, while this study has the value at 12.897 GgC/Kgwaste for the dry season and 12.547 GgC/Kgwaste for the wet season. However, the low value of DOC from Poland is evidence that developed countries have less degradable waste in their waste stream when compared to developing countries.

### Waste decomposition impact on methane generation

Sandoval-Cobo et al.^31^ stated that fresh waste disposed of produces more biodegradability and methane generation potential than excavated waste. However, methane generation is assumed to start immediately after the deposition of the waste, but in reality, this is not the case. A time lag of 2 to 6 months is considered after which the normal first-order decay process starts^32^. This explains why the value of methane generated during the study period is low. The degradable organic carbon is consumed slowly by the bacteria throughout a period at negative exponential rates, reflected in the process by which methane is generated and eventually emitted to the atmosphere^33^. However, the result for the DDOC decomposed in the disposal site was observed to have a quantity higher during the wet season likewise to methane generated in SWDS as stated by Dasgupta et al^19^. ^34^also revealed the effect of season on the decay rate (K) value that results to degradation.

### Methane generation based on seasonal variation

The FOD Model adopted in this study, as explained by Oonk^35^ considers CH_4_ generation from organic matter, which is assumed to progress in a complex order of reaction steps. First, the enzymes break apart the solid organic macro molecules into smaller one that is further processed by a micro-organism that leads to CH_4_ generation^25^. The CH_4_ emission process involved stages in which waste generators disposed of the waste into approved waste collection bins in designated locations within the city metropolis. After that, the waste collection trucks collect the waste into trucks and disposed of SWDS where they are decomposed after scavengers have picked the valuable materials for recovery and recycling. This follows the steps involved in MSW management such as waste generation, storage, collection, processing, and disposal as stated by IPPC and Maiyaki et al^2,36^. According to Kaza et al.^37^, the amount, complexities, and characteristics of generated waste depend on urbanization, population growth, economic prosperity, and improved standard of living. Other socio-economic factors identified to influence the MSW generation are: culture, tradition, attitudes, family life, and education according to Oluwalana et al^9^. However, this study has provided information that confirms that seasonal variation is also a factor that can affect the quantity of CH_4_ generation based on the degradability of the organic waste^38^.This study has the total value of methane generated as 0.465 Gg. Similar results generated in Ontario^33^ using Mass Balance models showed methane emissions of 0.74KgCO_2_Eq/KgMSW and 0.39KgCO_2_Eq/KgMSW. In Chennai City, India, the results of methane emission using a close flux chamber were in the range of 6.1 Gg/yr–9.1 Gg/yr, and 8.8 Gg/yr–11.3 Gg/yr, according to Srinivasan et al^16^. In Poland^,24^ used the First Order Decay Model and the Methane Generation Potential was 0.018 GgCH_4_/Ggwaste however, the estimated CH_4_ emission from the Landfill was 0.393 Gg/yr. But, the CH_4_ generated for this study during the dry season was 0.134 Gg while the value generated during the wet season was 0.331 Gg which is a 42.2% increase higher than the value obtained for the dry season. This variation could be explained as follows; A higher value than the 56 percent of the DOC as stated in the guidelines by IPCC is been converted to CH_4_ and CO_2_ during the periods when the temperature is < 20 °C^39^. Then, when the temperature becomes > 20 °C it leads to low precipitation, resulting in unintentional and uncontrollable fire outbreaks in SWDS, a frequent incidence during the dry season. Jain et al.^40^ in the research conducted in the United States, investigated waste composition kinetics for closed landfills and assumed that a fraction of the in-place biodegradable waste may not be decomposing, potentially due to a lack of adequate moisture content during the dry season, the results showed that the decay rate is significantly correlated with annual precipitation, It does not correlate with per capita income or lifestyle as stated by Akintayo and Olanisakin^11^. Therefore, methane generated is equal to methane emitted because there is no methane recovery for energy purposes. Also, the SWDS are unmanaged with a shallow depth of < 5 m. Hence, the oxidation is zero. This poor waste management practice requires an increased public awareness about the effect, so that they would be improved perception and disposition towards sustainable MSW Management approach as stated by Adekola et al^41^. Thereby considering the relocation of all the dumpsites located in the mainland (Fig. 1) to outskirt of the city metropolis. Finally, Food waste, Grasses/Garden and Wood waste quantities in SWDS depends on seasonal variation while Paper/Textile waste does not.

## Conclusion

This study conducted waste characterization from four major SWDSs in Lagos and the results showed that all the sites generated similar waste types at different quantities per season. Also, the DOC values for both seasons for the degradable waste were estimated based on the percentage of organic waste in the waste stream and finally, the methane gas emitted was determined based on seasonal variations and it was revealed that the wet season generated more methane gas than the dry season. This confirmed that the organic fraction of MSWthat produces CH4 gas also depends on the seasonal conditions. Furthermore, the study has also provided State-specific data on MSWgenerated in Lagos. Therefore, the organic fraction of MSW, which causes methane generation, should be segregated from the non-biodegradable component at the point of waste generation. Hence, biomass generation for compost and resource recovery for recycling/reuse is recommended. Further research is therefore recommended to provide other states-specific data in Nigeria that can be scaled up to country-specific activity data for national greenhouse gas inventory purposes, which is currently unavailable, Also, a projected duration of the study is required to cover a time lag of 1–5 years.

## Data Availability

All data generated or analyzed during this study are included in this published article.
